# Leu128^3.43^ (L128) and Val247^6.40^ (V247) of CXCR1 Are Critical Amino Acid Residues for G Protein Coupling and Receptor Activation

**DOI:** 10.1371/journal.pone.0042765

**Published:** 2012-08-24

**Authors:** Xinbing Han, Souvenir D. Tachado, Henry Koziel, William A. Boisvert

**Affiliations:** 1 Department of Medicine, Beth Israel Deaconess Medical Center, Harvard Medical School, Boston, Massachusetts, United States of America; 2 Department of Vascular Medicine, Brigham and Women's Hospital, Harvard Medical School, Cambridge, Massachusetts, United States of America; 3 Center for Cardiovascular Research, John A. Burns School of Medicine, University of Hawaii, Honolulu, Hawaii, United States of America; Louisiana State University, United States of America

## Abstract

CXCR1, a classic GPCR that binds IL-8, plays a key role in neutrophil activation and migration by activating phospholipase C (PLC)β through Gα_15_ and Gα_i_ which generates diacylglycerol and inositol phosphates (IPs). In this study, two conserved amino acid residues of CXCR1 on the transmembrane domain (TM) 3 and TM6, Leu128^3.43^ (L128) and Val247^6.40^ (V247), respectively, were selectively substituted with other amino acids to investigate the role of these conserved residues in CXCR1 activation. Although two selective mutants on Leu128, Leu128Ala (L128A) and Leu128Arg (L128R), demonstrated high binding affinity to IL-8, they were not capable of coupling to G proteins and consequently lost the functional response of the receptors. By contrast, among the four mutants at residue Val247 (TM6.40), replacing Val247 with Ala (V247A) and Asn (V247N) led to constitutive activation of mutant receptors when cotransfected with Gα_15_. The V247N mutant also constitutively activated the Gα_i_ protein. These results indicate that L128 on TM3.43 is involved in G protein coupling and receptor activation but is unimportant for ligand binding. On the other hand, V247 on TM6.40 plays a critical role in maintaining the receptor in the inactive state, and the substitution of V247 impaired the receptor constraint and stabilized an active conformation. Functionally, there was an increase in chemotaxis in response to IL-8 in cells expressing V247A and V247N. Our findings indicate that Leu128^3.43^ and Val247^6.40^ are critical for G protein coupling and activation of signaling effectors, providing a valuable insight into the mechanism of CXCR1 activation.

## Introduction

Interleukin-8 (IL-8) is a member of the CXC-chemokine family and is a potent chemotactic factor for neutrophils [1] and natural killer cells [2]. IL-8 activates these cells via two related chemokine receptors CXCR1 and CXCR2 [3], [4], [5], [6]. Although both receptors bind IL-8, CXCR1 and CXCR2 have distinct physiological activities. CXCR1 is generally more resistant to desensitization and downregulation [3], and is also important in the generation of antimicrobial responses and in the respiratory burst upon neutrophil activation [3]. Inflammatory diseases such as chronic obstructive pulmonary disease (COPD), asthma, inflammatory bowel diseases, and Crohn's disease are thought to be exacerbated by neutrophils. As such, targeting CXCR1 using structural and biochemical means to design specific antagonists is a promising therapeutic strategy to modulate the activity of the receptor to combat these diseases [7], [8], [9], [10], [11], [12]. Additionally, because CXCR1 promotes IL-8-mediated tumor growth, CXCR1 blockade can selectively target and eliminate human breast cancer stem cells [13], androgen-independent prostate cancer [14], [15], and malignant melanoma [16], [17], highlighting IL-8/CXCR1 signaling as a possible therapeutic intervention point in targeting the tumor microenvironment [18].

CXCR1 is a member of GPCRs that feature the characteristic seven transmembrane domains. Upon activation, CXCR1 couples to both pertussis toxin-sensitive Gα_i_ and pertussis toxin-resistant Gα_15_ [19] to mediate CXCR1-activated signal transduction pathways. These pathways are necessary for the induction of inflammatory responses or for more subtle regulation of cellular functions such as phosphoinositide (PI)_3_ hydrolysis, intracellular Ca^2+^ mobilization, and chemotaxis [20]. Several critical amino acid residues and functional motif/domains of the human CXCR1 have been identified, including the N terminal region responsible for determining the receptor subtype selectivity [21] and receptor activation [22], as well as the C terminal tail which is involved in IL-8-induced internalization [23], migration and activation [4], [20]. Some residues of CXCR1 involved in agonist binding, signaling activation and receptor internalization have been identified [24], [25], [26], [27]. Despite these substantial advances, however, the exact mechanism of chemokine receptor activation is still largely unknown.

GPCRs possess similar structures that consist of seven transmembrane helices containing well-conserved sequence motifs, which suggests that they are probably activated by a common mechanism [28], [29], [30]. Among the seven TMs, TM3 and TM6 are thought of as switches for GPCR's activation. These domains play an important role for the transition to their fully activated state [29], [31], [32] and in interactions between the receptor and G proteins [33]. Analysis of crystal structure of rhodopsin and agonist-bound human adenosine A2A receptor suggests that activation of GPCRs is due to disruption of key interhelical contacts [33], [34]. This activation involves the rotation of TM3 and TM6 domains and affects the conformational structure of G protein-interacting cytoplasmic loops of the receptor, thereby uncovering previously masked G protein-binding sites on the intracellular loops [31].

Within the structure of GPCR, Leu128^3.43^ (superscript in this form indicates Ballesteros–Weinstein numbering for conserved GPCR residues), located at the region near the second intracellular loop in TM3, is highly conserved (over 70%) [35] which suggests that this residue may play an important structural and functional role in other GPCRs as well. Based on CXCR1 homology with the crystal structure of rhodopsin, Val 247 is predicted to lie near the cytoplasmic end of TM6, which is oriented towards several highly conserved amino acids in TM2, TM3 and TM7. As rhodopsin and other type A GPCRs may share a common mechanism of receptor activation [28], [29], [30], we investigated the role of L128 in TM3.43 and V247 in TM6.40 in G protein coupling and signaling properties of constitutively active forms of the human CXCR1.

It is noteworthy that IL-8 and its specific receptor CXCR1 are identified in humans but are not found in the traditional rodent model organisms. As a consequence, we utilized well established cell models which were transfected with CXCR1 and other related cellular components to study the functions of IL-8 and CXCR1 as well as the mechanisms involved in receptor activation by IL-8. In this study, we generated selective mutations on two conserved amino acid residues of CXCR1 on TM3 and TM6, Leu128^3.43^ (L128) and Val247^6.40^ (V247), respectively. We analyzed their features including receptor expression, and ligand binding. The capacity of Gα_15_ and Gα_i_ to be coupled by CXCR1 mutants is determined by measuring downstream signaling transduction pathways such as PLCβ-IP_3_ production and cAMP production. To test for functional consequence of activation by CXCR1 mutants, we performed chemotaxis assays with cells that express CXCR1 and its mutants.

## Materials and Methods

### Construction of Expression Vectors and Site-directed Mutagenesis

Wild type (WT) human CXCR1 cloned into pSFFV•neo vector was provided as a gift from Professor Ingrid Schraufstatter at the La Jolla Institute for Experimental Medicine. After digestion with EcoRI, wild type human CXCR1 fragment was subcloned into the pSG5 vector. Oligonucleotides for site-directed mutagenesis were designed to yield several different amino acid substitutions and were synthesized by GIBCO BRL. CXCR1 mutations were generated with the Transformer mutagenesis kit (Clontech, Palo Alto, CA) and were confirmed by Big Dye Terminator Cycle Sequencing (Perkin Elmer) of the final construct. Plasmid DNA for transient transfection was purified with the EndoFree Plasmid Maxi Kit from Qiagen.

### Transient Transfection

COS-7 cells and human embryonic kidney 293 cells (HEK293 cells) from the American Type Culture Collection (ATCC, Rockville, MD) were maintained at 37°C in humidified air containing 5% CO2 in DMEM with 10% FBS. Cells were grown to 60∼80% confluency prior to transient transfection. Transfection was performed using LipofectAMINE reagent (GIBCO BRL, Gaithersburg, MD) according to the manufacturer's instruction. COS-7 cells were incubated with the transfection complex for 5 hrs at 37°C. After removal of the transfection medium, the cells were incubated in DMEM with 10% FCS overnight.

### Flow Cytometry

HEK 293 cells were transfected with either pSG5 plasmid, or CXCR1 WT or mutants. After 48 hr, cells were dissociated and fixed with 4% paraformaldehyde. Cells were then washed three times with cold staining buffer before incubation with mouse anti-human CD128 FITC (CXCR1) from BD Pharmingen (Cat# 555939) (San Diego, CA) on ice for 20 min. Cells were washed three times and resuspended in 0.5 ml of staining buffer. Stained cell samples were analyzed by flow cytometry [36].

### Confocal microscopy

COS-7 cells or TSA-201 cells (# 96121229 from Sigma-Aldrich, St Louis, MO) were transfected with either pSG5 plasmid, or CXCR1 WT or mutants. After 24 hr, cells were fixed with 4% paraformaldehyde followed by blocking with 1% BSA in HBSS. Cells were incubated with primary antibody (mouse anti human CD128a (CXCR1)) from BD Pharmingen (5A12 clone) (San Diego, CA) at 4°C overnight. After washing three times, the cells were incubated with the secondary antibody (Alexa Fluor 488, goat anti-mouse IgG(H+L) from Invitrogen, CA, or DyLight 549-conjugated goat anti-mouse IgG(H+L) from Jackson ImmunoResearch Laboratories, Inc., PA) at RT for 1 hr and counterstained with Dapi. Expression of CXCR1 or the mutants was observed using confocal microscope.

### IL-8 Binding Assay

COS-7 cells were plated in 12-well plates at a density of 1.6×10^5^ cells/ml of DMEM/10% FCS and incubated overnight. The cells were transfected as described before. In brief, the cells were transfected with 0.7 µg DNA plus 2.5 µl of LipofectAMINE reagent and 5 µl Plus Reagent (total transfection volume was 0.5 ml/well). After 48 hrs from the start of transfection, transfected cells were washed in the binding buffer (HBSS medium containing 0.5% BSA and 25 mM HEPES buffer). The cells were then incubated for 2 hr at 4°C. The final concentration of ^125^I-IL-8 in the 0.5 ml/well (for 12-well plate) of medium was 0.07 nM (0.05 µCi). The range of unlabeled IL-8 concentrations in the binding assays was 0.1–300 nM [0, 0.1, 0.3, 1, 3, 10, 30, 100, 300 nM]. The nonspecific ^125^I-IL-8 binding was determined by incubating cells with ^125^I-IL-8 in the presence of 250 nM of cold IL-8. In experiments designed to test the maximal binding of ^125^I-IL-8 to CXCR1 WT and mutants, unlabelled IL-8 was not added. After washing three times with 2 ml of ice-cold binding buffer, the cells were lysed in 0.5 ml of 1 N NaOH and the lysates were counted using a scintillation counter. The data were curve fitted and affinity constant (Kd) and maximum total binding (B_max_) were calculated using GraphPad Prism (San Diego, CA). All experiments were carried out in triplicate.

### Inositol Phosphate (IP) Assays

For IP assay, COS-7 cells were plated in 24-well plates with a density of 1×10^5^ cells per well the day before transfection. The cells were cotransfected with 0.3 µg CXCR1 plasmid DNA (or its mutants) and 0.3 µg Gα_15_ plus 1.5 µl LipofectAMINE reagent and 0.5 µl Plus reagent. In experiments of cotransfection with WT CXCR1 or its mutants, COS-7 cells were cotransfected with equal amounts of cDNA (0.1 µg per well per component). Plasmids encoding Gα_i2_ and PLCβ_2_ were generous gifts from Dr. Kozasa at the University of Illinois at Chicago. Plasmids encoding Gβ_1_ and Gγ_2_ were kindly provided by Guthrie Research Institute, PA. (Sayre, PA). 24 hr after transfection, cells were incubated with inositol-free medium containing 2 µl/ml myo-[2-^3^H]inositol (Dupont-NEN, Boston, MA) in the absence or presence of 100 ng/ml pertussis toxin (PTX) as indicated for 18 hr. At 48 hr from the start of transfection, cells were washed with assay buffer [Hank's balanced salt solution, 0.5% (w/v) crystalline bovine serum albumin, 20 mM HEPES-NaOH, pH7.4]. IP production was measured by incubating the cells for 1 hr at 37°C in 0.2 ml assay buffer containing 10 mM LiCl in the presence or absence of 40 nM IL-8. Perchloric acid was added to each well and samples were then neutralized with a solution of 0.72 M KOH and 0.6 M KHCO3 before being centrifuged. Total IP was measured using Dowex AG1-X8 anion exchange column chromatography (Bio-Rad, Richmond, CA). All assays were performed in triplicate, on at least 3 separate occasions with different batches of cells, and always included control cells transfected with WT CXCR1. Data were analyzed using GraphPad Prism (San Diego, CA) and expressed as fold-increase over basal conditions in cells cotransfected with WT CXCR1 and Gα_15_ plasmid.

### Intracellular calcium mobilization assays

COS-7 cells were transiently transfected with CXCR1 WT or V247N mutant. Preparation of dye-loading solutions of BD calcium assay kits (BD Biosciences, Rockville, MD.) were performed and fluorescence was measured as described in the manual.

### cAMP assay

How CXCR1 WT and V247N mutant would affect cAMP formation was assessed in both COS-7 and HEK-293 cell-derived TSA-201 cells. Both cell lines are useful to demonstrate GPCR-triggered cAMP accumulation. However, unlike COS-7 cells, type II adenylyl cyclase I in TSA-201 cells is not stimulated by free βγ subunits released from activated Gα_i_ protein which can inhibit cAMP accumulation when transfected with Gα_i_-coupled receptors such as CXCR1 [37], [38]. COS-7 cells and HEK293 cell-derived TSA-201 cells were maintained in DMEM, and supplemented with 10% FBS. COS-7 cells were transiently transfected with 0.2 µg of CXCR1 or CXCR1 mutant V247N as well as Gα_i2_, Gβ_1_, and Gγ_2_. TSA-201 cells were grown to 50% confluency in DMEM/10% FBS on 24-well plates and were transfected using lipofectaMINE with 0.2 µg of each DNA encoding human LH receptor, or CXCR1 WT, or V247N mutant per well. 48 hours after transfection, COS-7 cells or TSA-201 cells were washed once with 1×HBSS/0.1% BSA pre-warmed buffer. The cells were incubated with 1×HBSS/0.1% BSA containing 0.5 mM IBMX for 30 min followed by treatment of the cells with the same medium containing either forskolin (10 µM) or hCG (10^−7^ M) in the presence or absence of IL-8 (40 nM) for another 30 min. Then the treatment buffer was aspirated and the reaction stopped with 0.85 ml of 5% cold perchloric acid (PCA). It was left for 10 min before the transfer of 0.8 ml to the 12×75 glass tubes with 0.18 ml of 3× neutralization buffer (0.72 M KOH, 0.6 M KHCO3). The samples were vortexed and centrifuged at 2,500× g for 5 min. cAMP levels were measured with a RIA Kit (Biomedical Technologies Inc., Stoughton, MA) according to the manufacturer's instructions. Data are shown as mean ± SEM for three assays with each condition performed in triplicate.

### Chemotaxis assay

IL-8 stimulated chemotaxis through CXCR1 and CXCR1 mutants was assessed using a 48-well microchemotaxis chamber technique (Neuro-Probe, Gaithersburg, MD). CXCR1 or CXCR1 mutant V247A or V247N as well as Gα_i_, Gβ, Gγ, and PLCβ_2_ was transiently transfected in HEK293 cells. 24 hours after transfection, the cells were serum-starved in 0.5% FCS-containing medium for another 24 h. The cells were then trypsinized, treated with trypsin inhibitor (Sigma), resuspended in BSA medium at 5×10^5^ cells/mL, and incubated for 2 h at 37°C. To the bottom wells 28 µl of either 100 ng/ml IL-8 or the solution without IL-8 (control group) were added as chemoattractant. For some indicated wells, cells were pretreated with 200 ng/ml of pertussis toxin for 10 min before being added to the upper chamber for chemotaxis assay. For all the migration assays, 50 µl of 1×10^6^ cells/mL were loaded into the upper chamber fitted with a 10-µm pored membrane (Nucleopore, Corning Costar, Acton, MA) coated with 50 µg/mL rat collagen type 1 (Collaborative Biomedical Products, Bedford, MA) for 4 h. This allowed cells to migrate towards the underside of the insert filter. Cells that did not migrate through the pores and therefore remained on the upper side of the filter membrane were gently removed with a cotton swab. Cells on the lower side of the insert filter were quickly fixed with 5% glutaraldehyde solution for 10 minutes and then stained with 1% Crystal Violet in 2% ethanol for 20 minutes. The cells that adhered to the lower surface of the membranes were counted microscopically at 400× magnification. The chemotactic activity was expressed as a chemotactic index, calculated as the number of cells that migrated in response to IL-8, divided by the number of cells that migrated toward the negative control (dilution buffer without IL-8 for control cells). Statistical analysis was performed using Student's t test.

## Results

### Construction and expression of CXCR1 mutants

**Figure 1:**
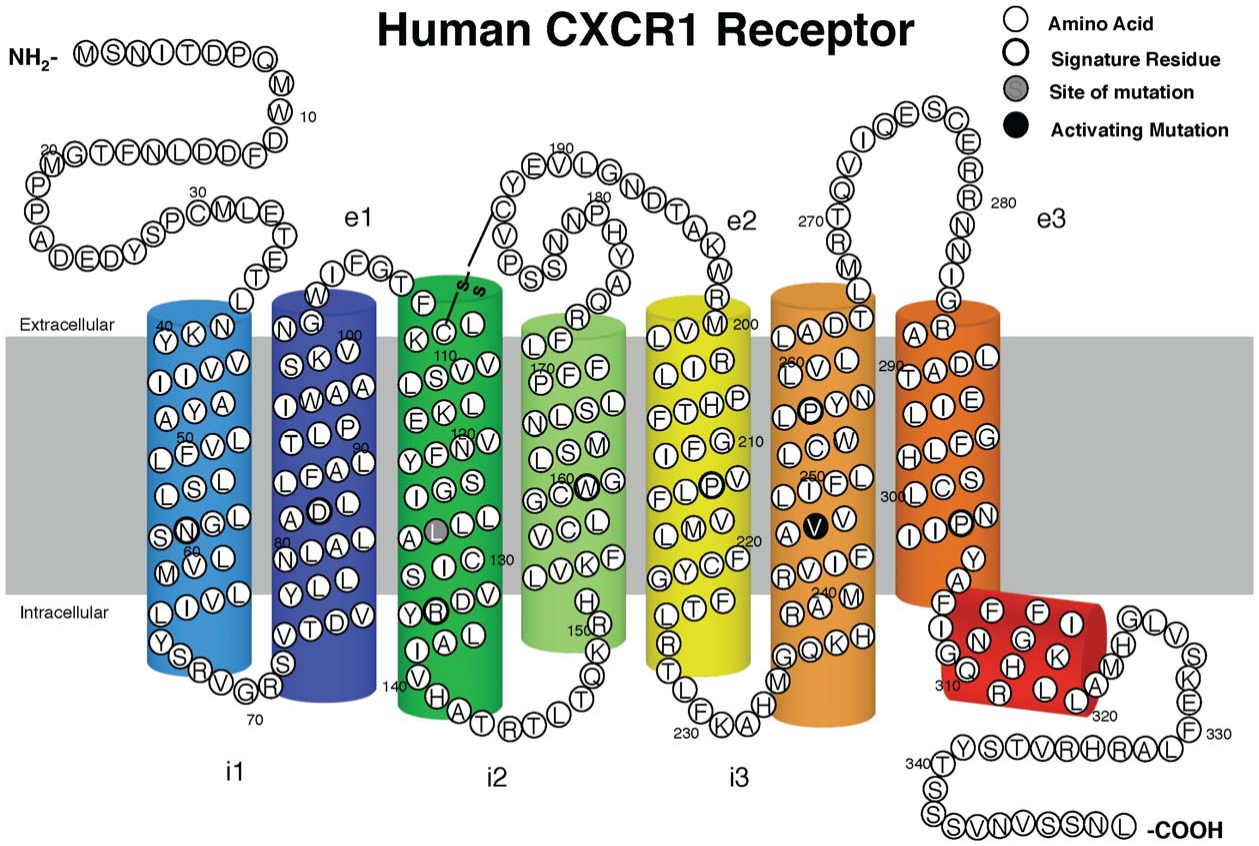
Two-dimensional diagram of human CXCR1 receptor. The positions of the residues (Leucine 128 and Valine 247) that were targeted for mutagenesis in TM3 and TM6 of the CXCR1 are indicated by gray and black filled circles respectively. The putative disulfide bridge, formed by Cys110/Cys 187 is indicated by “S-S”.

To define the G protein coupling and signaling properties of constitutively active forms of the human CXCR1, we generated 7 different mutations at 2 residues that correspond to known sites of activating mutations in TM3 and TM6 of rhodopsin, the human LHR, and several other GPCRs [39], [40], [41], [42], [43], [44], [45]. Three amino acid substitutions of the L128 (L128A, L128Q, and L128R) and four amino acid substitutions of the V247 (V247A, V247F, V247N and V247Y) in the chemokine receptor CXCR1 were made using mutagenesis to analyze their functional role (Fig. 1).

**Figure 2:**
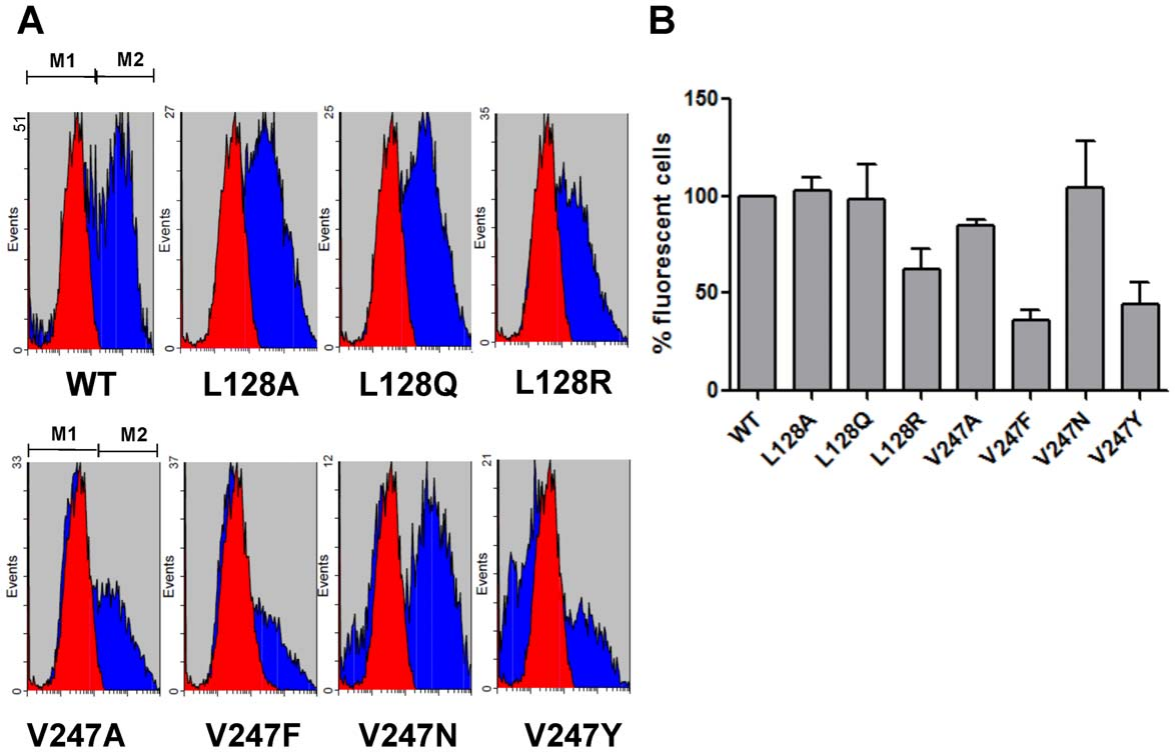
FACS analysis of CXCR1 WT and mutants. HEK 293 cells were transiently transfected with CXCR1 or its mutants. Cells were incubated with FITC-conjugated mouse anti-human CD181 (CXCR1) antibody. Specificity of signal was confirmed by staining the cells with mouse IgG1 isotype control. (A) Transfected HEK 293 cell population stained positive for this anti-CXCR1 antibody (M2 region). (B) Fluorescence of positively stained cells was quantified by FACS (± SEM, with CXCR1 WT set to 100%).

Flow cytometry (Fig. 2) utilized to monitor the expression of CXCR1 and its mutants showed that WT and mutant CXCR1 were expressed on transfected cells (Fig. 2 A, B). The expression pattern was confirmed also by confocal imaging (data not shown).

### Ligand binding assay of CXCR1 mutants

**Figure 3:**
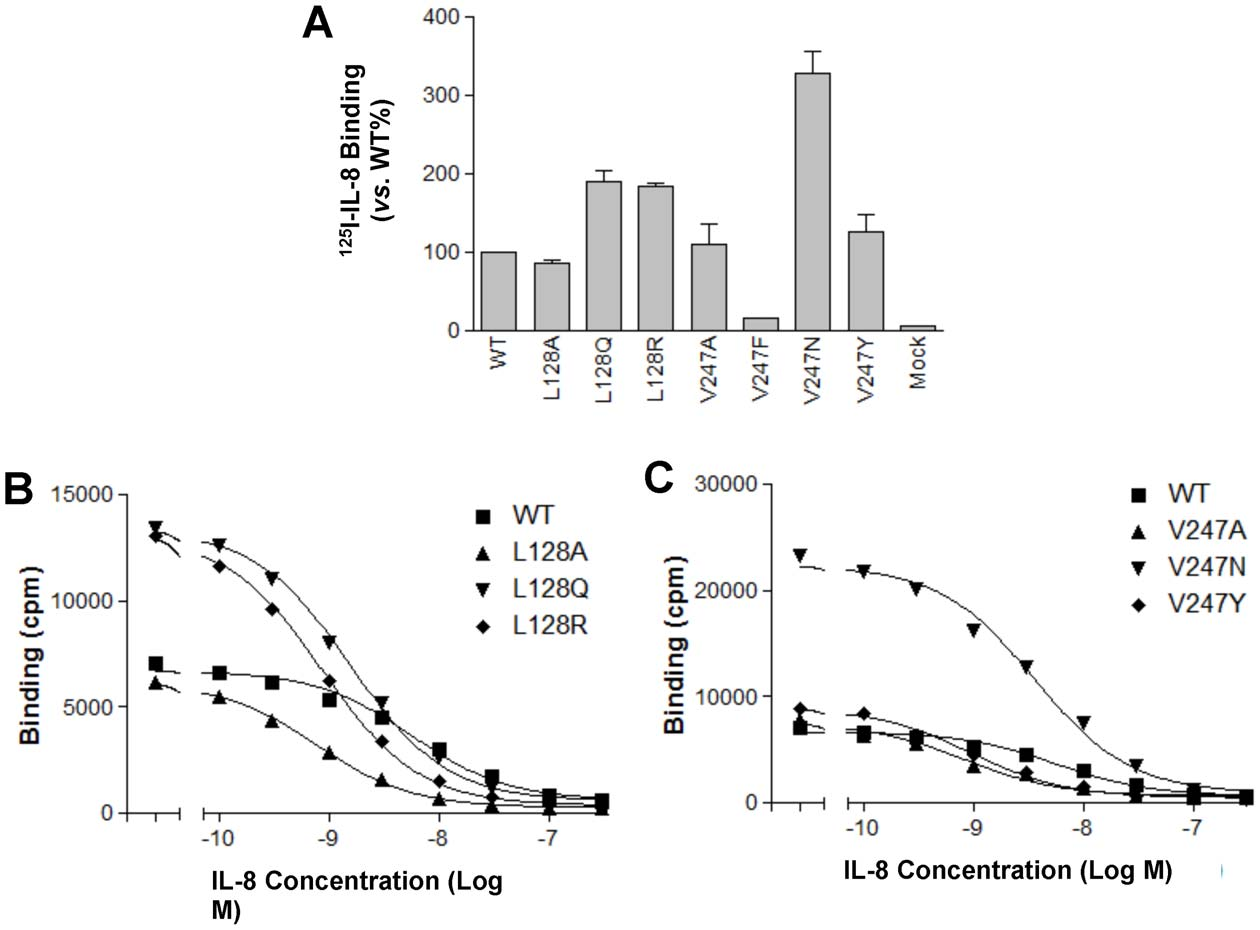
Binding of ^125^I-labeled IL-8 to COS-7 cells expressing WT CXCR1 or its mutants. The CXCR1 mutants were transiently transfected in COS-7 cells. Maximal binding of ^125^I-IL-8 to CXCR1 WT and mutants (A) and competition binding experiments (B and C) were performed as described under “Materials and Methods”. Competitive binding studies were conducted using ^125^I-labeled IL-8 and various unlabelled ligands as described under “Materials and Methods”. Representative maximal binding experiments for L128 and V247 mutants are shown in Fig. 3A, performed in triplicates. Mock was transfected with pSG5 plasmid. Representative competitive binding experiments for L128 and V247 mutants are shown in Fig. 3(B) and Fig. 3(C), performed in triplicates. Nonspecific binding was determined by adding 250 nM unlabeled IL-8. Curve fitting was done using GraphPad Prism data analysis program, and the affinity constant (K_d_) for CXCR1 mutants is shown in Table 1.

We performed ligand binding assay determine if the CXCR1 mutants are capable of binding to specific ligands, and to assess the maximum binding number (Fig. 3A) and affinity constant (K_d_) of the mutant receptors (Fig. 3 B and C). Mock (transfected with pSG5 vector) did not show ^125^I IL-8 ligand binding, suggesting that there is no endogenous IL-8 receptor such as CXCR1 expression in COS-7 cells (Fig. 3A). Among mutants detected, V247F barely bound to ^125^I IL-8 ligand whereas all other CXCR1 mutants showed specific binding to ^125^I IL-8 ligand (Fig. 3 A, B and C). The results of ligand binding assays for CXCR1 mutants are summarized in Table 1. The affinity constant (K_d_) of WT CXCR1 for IL-8 obtained by ligand binding assay in transfected COS-7 cells was 4.87×10^−9^ M, which was similar to those reported by others both in recombinant cell lines and in neutrophils [46], [47]. It was noteworthy that mutants L128Q and V247N showed K_d_ values similar to the WT, while other mutants including L128A, L128R, V247A and V247Y, demonstrated increased K_d_ values (Table 1). The altered K_d_ values from the mutants suggested that the receptor exists in multiple conformations.

**Table 1:** Summary of ligand binding assay of CXCR1 WT and mutants^1^.

CXCR1	Location	CXCR1 &	B_max_	K_d_ (M)
Residue	(Baldwin#)	Mutants	(% of WT)	
		WT	100±0	4.87e-09
Leu 128	TM3, residue 43	L128A	86.8±2.8	5.35e-10
		L128Q	189.4±15.5	1.35e-09
		L128R	184.1±3.0	6.86e-10
Val 247	TM6, residue 40	V247A	109.9±26.0	5.50e-10
		V247F	N.D.	N.D.
		V247N	328.5±26.7	3.14e-09
		V247Y	126.2±21.1	6.93e-10

1 B_max_ and K_d_ value were estimated from COS-7 cells ligand binding assay as described under “Materials and Methods”. All assays were done in triplicates. Values are mean ± SEM from 3 experiments.

### Specific mutation on TM6 (V247) of CXCR1 leads to Gα_15_ coupling and constitutively activated inositol phosphate accumulation

**Figure 4:**
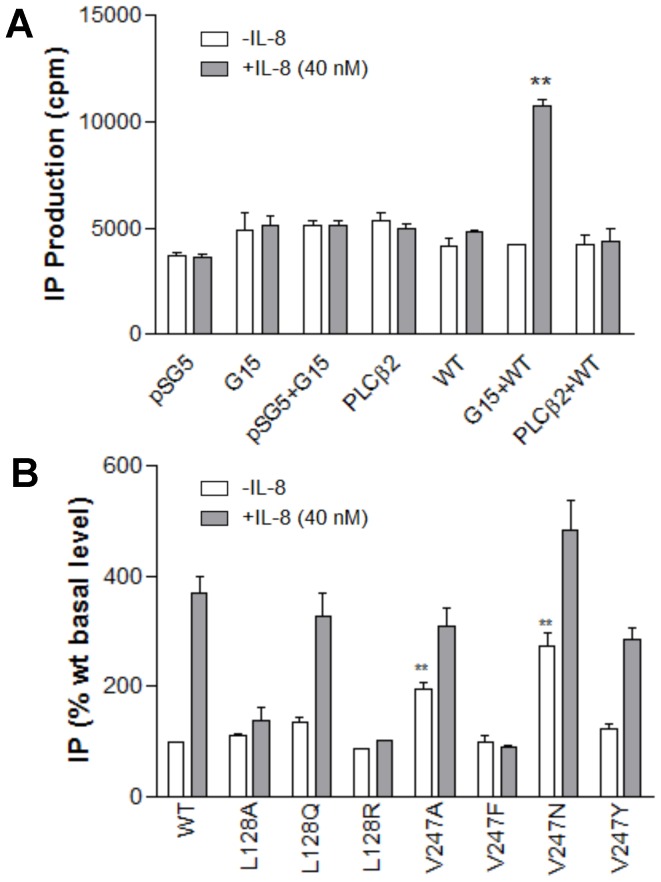
CXCR1 mutants coupled to Gα_15_. (A) Activation of PLCβ_2_ by CXCR1 through Gα_15_ proteins. COS-7 cells were cotransfected with equal amounts of cDNA (0.3 µg per well per component) encoding either pSG5, PLCβ_2_, Gα_15_, CXCR1 wild type (WT), Gα_15_ plus WT, or PLCβ_2_ plus WT. Inositol phosphates were measured 1 hours after treatment in the presence (shaded bars) or absence (open bars) of IL-8 (40 nM). Data are mean ± SEM of replicate wells. ** *P*<0.01, Gα_15_+WT (+IL-8) *vs*. Gα_15_+WT (−IL-8). (B) CXCR1 mutants coupled to Gα_15_. Basal and IL-8-stimulated inositol phosphate (IP) accumulation in COS-7 cells transiently co-transfected with WT (0.3 µg) or mutant CXCR1 (0.3 µg) and Gα_15_ (0.3 µg). Data for mutants are summarized from 3–7 experiments, each performed in triplicate, and are expressed as a percentage of WT CXCR1 baseline determined in parallel. The results are mean ± SEM. ** *P*<0.01, *vs*. WT+ Gα_15_ (in the absence of IL-8).

Gα_15_ protein is expressed exclusively in hematopoietic cells of myeloid lineage on which CXCR1 is expressed. The Gα_15_ activates the phospholipase Cβ isoform in a PTX-resistant manner and causes inositol phosphate accumulation [48]. For these studies, we used COS-7 cells and HEK-293 cells to take advantage of the lack of endogenous CXCR1 expression with the added convenience of transfection with high efficiency. Both cell lines have been well established as cell models to study CXCR1 and its signaling [19], [25], [26]. In this study, IL-8 treatment of cells transfected with WT CXCR1 alone did not stimulate IP formation as shown in Fig. 4A. This suggested that CXCR1 was coupled to neither Gα_q_ nor Gα_s_ present in COS-7 cells. In fact, none of the CXCR1 mutants could stimulate PLCβ unless they were coexpressed with Gα_15_. Cotransfection with mock pSG5 and Gα_15_ had no effect on IP formation in response to IL-8, suggesting that there was no endogenous CXCR1 present in COS-7 cells (Fig. 4A). COS-7 cells did not have any measurable PLCβ response to IL-8 in the absence of Gα_15_ expression, indicating that there was insufficient PLCβ_2_ or PLCβ_3_ expressed for a Gα_i_-dependent, βγ-mediated IL-8 response. As shown in Fig. 4A, the PLCβ response could be reconstituted by expressing Gα_15_.

To examine the role of L128 and V247 in Gα_15_ coupling, CXCR1 mutants were cotransfected with Gα_15_ in COS-7 cells. While mutant L128Q behaved like WT with regard to PLC activation, mutant L128A and L128R were not effective in IP accumulation in response to IL-8 (Fig. 4B). This suggested that introduction of A or R for L128 abolished IP accumulating property, implying the important role of L128 in Gα_15_ coupling. It is noteworthy that two mutants, V247A and V247N were able to elevate basal levels of IP in transfected COS-7 cells by 196% and 272%, respectively, in the absence of IL-8. IL-8-independent activation of the phospholipase C signaling pathway indicated that both V247A and V247N are constitutively active mutants coupled to Gα_15_ resulting in PLCβ activation and IP accumulation.

**Figure 5:**
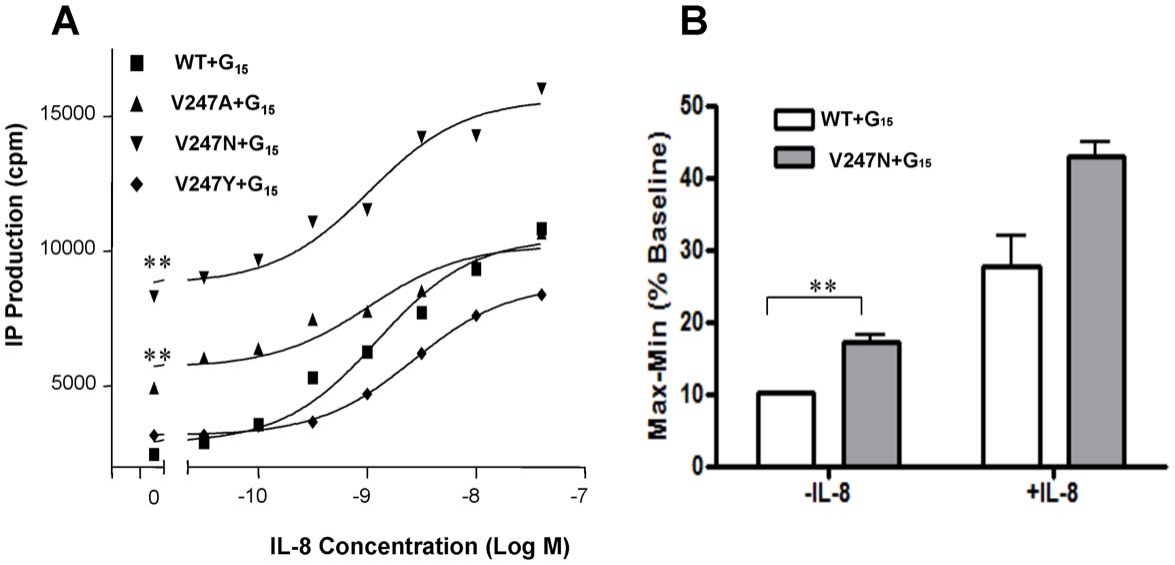
Constitutively active mutants of CXCR1. (A) Basal and IL-8-stimulated inositol phosphate (IP) accumulation in COS-7 cells transiently co-transfected with WT or mutant CXCR1 and Gα_15_. IP production was determined in detail as described under “Materials and Methods”. Data are mean ± SEM of replicate wells from a representative experiments performed in triplicate. **, *P*<0.01, *vs*. WT+ Gα_15_ (in the absence of IL-8). (B) Calcium signaling capacity in COS-7 cells transfected with CXCR1 WT and V247N mutant. The increase in intracellular calcium concentration ([Ca^2+^]_i_) in COS-7 cells transfected with Gα_15_ and CXCR1 WT or V247N mutant was measured using BD calcium assay kit. The cells were stimulated with or without IL-8 (40 nM). Values represent the mean (± the SEM) increase of [Ca^2+^]_i_ (*n* = 4). **, *P*<0.01, *vs*. WT+ Gα_15_ (in the absence of IL-8).

To assess whether V247 mutants display unique features and to determine the degree of IP production between A and N mutants, two constitutively active CXCR1 mutants were cotransfected with Gα_15_ in COS-7 cells followed by treatment with varying concentrations of IL-8 (Fig. 5A). In the absence of IL-8, both V247A and V247N were coupled to Gα_15_ and constitutively activated the IP accumulation. In response to IL-8, however, the degree of IP production was dissimilar between A and N. Although both V247A and V247N stimulated IP accumulation in response to IL-8 in a dose-dependent manner in COS-7 cells, the maximum IP accumulation of V247A in response to IL-8 was distinct from that of V247N. The V247A showed maximum IP accumulation similar to WT in response to 40 nM of IL-8, whereas IP accumulation of V247N in response to IL-8 (40 nM) was much higher than WT (Fig. 5A). As expected, intracellular calcium changes were more robust in V247N transfected cells than CXCR1 WT transfected cells (Fig. 5B).

### Ability of CXCR1 WT and mutants to activate Gα_i_

**Figure 6:**
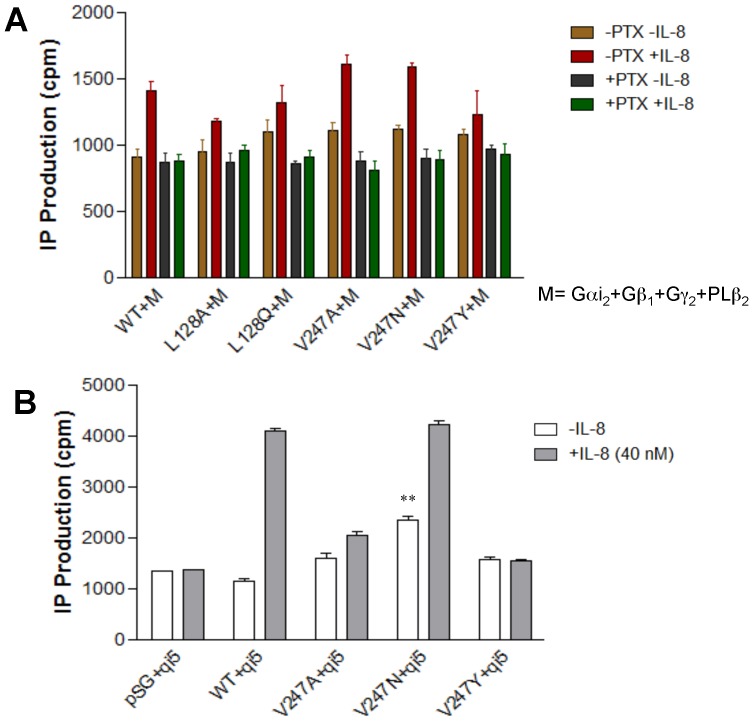
CXCR1 and mutants coupled to Gα_i_. (A) COS-7 cells were cotransfected with equal amounts of cDNA (0.1 µg per well per component) encoding Gα_i2_, Gβ_1_, Gγ_2_, PLCβ_2_, the WT CXCR1 or its mutants. (B) COS-7 cells were cotransfected with equal amounts of encoding Gα_qi5_ (qi5) and the WT CXCR1 or its mutants. The release of inositol phosphates, induced by 40 nM IL-8, was measured 1 hour after the treatment in the presence or absence of pertussis toxin (PTX) (100 ng/ml) for 18 hrs. Data are mean ± SEM of replicate wells from a representative experiment. **, *P*<0.01, *vs*. WT+ qi5 (in the absence of IL-8).

CXCR1 could activate βγ subunits of endogenous Gα_i/o_ proteins independently of their coupling to recombinant Gα_15_ protein. To investigate the mechanism by which CXCR1 signaling is activated, CXCR1 WT or mutants were cotransfected into COS-7 cells with Gα_i2_, Gβ_1_, Gγ_2_ and PLCβ_2_. This five-component cotransfection system (Gα_i2_- Gβ_1_- Gγ_2_ - PLCβ_2_) has been successfully applied to investigate IL-8 signaling pathways in COS-7 cells in a previous study [19]. In this cotransfection system, Gα_i2_ activation in response to IL-8 leads to the release of Gβγ subunits from Gα_i2_, which results in PLCβ_2_ activation and subsequent IP accumulation [19]. As shown in Fig. 6A, mutants L128A, L128Q, as well as V247A, V247N and V247Y stimulated IP accumulation in response to IL-8, much like the WT CXCR1 counterpart. Furthermore, PTX, a specific inhibitor of Gα_i_, totally suppressed Gα_i_ activation and subsequent IP accumulation, suggesting that CXCR1 and its mutants investigated here are indeed coupled to Gα_i2_. Thus, IP accumulation could be achieved via the PTX-insensitive Gα_15_ pathway or the PTX-sensitive Gα_i2_ pathway as described above. Interestingly, L128R and V247F failed to have any effect on IP formation in response to IL-8 in transfected COS-7 cells (data not shown). Among all mutants detected, V247F bound the most poorly to IL-8. Because this mutant showed low expression as indicated by flow cytometry (Fig. 2A and B), it is likely that binding to IL-8 was low due to fewer mutant receptor expressed on fewer cells (Fig. 3A). Because L128R has high specific binding to IL-8, it is likely that the conformational changes of L128R impaired coupling to Gα_i2_ whereas the failure of V247F to respond to IL-8 resulted from the lack of ligand binding.

The coupling of CXCR1 and its mutants to Gα_i2_ was further supported by data shown in Fig. 6B. COS-7 cells were cotransfected with equal amounts of encoding Gα_qi5_ (qi5) and the WT CXCR1 or its mutants. The chimeric G protein, Gα_qi5_, contains main structure of Gα_q_ and the last five C-terminal amino acids of Gα_q_ were replaced with the corresponding amino acids from Gα_i2_. Mutation of the last five C-terminal amino acids is sufficient to completely switch receptor coupling selectivity and enable Gα_i/o_-coupled GPCR signaling through phospholipase C_β_ (PLC_β_). As such, a classical Gα_q_-mediated output becomes indicative of receptor coupling to Gα_i/o_ [49], [50]. Transient expression of Gα_qi5_ in the COS-7 cell line facilitated the activation of CXCR1 WT and V247N mutant in response to IL-8. Moreover, in the absence of IL-8, transfected cells showed significantly increased IP accumulation in V247N and Gα_qi5_ coinfected cells, suggesting that CXCR1 WT and V247N are coupled to Gα_i_ protein and that V247N is capable of constitutively activating Gαi protein (Fig. 6B).

### CXCR1 mutant V247N is capable of constitutively activating Gα_i_

**Figure 7:**
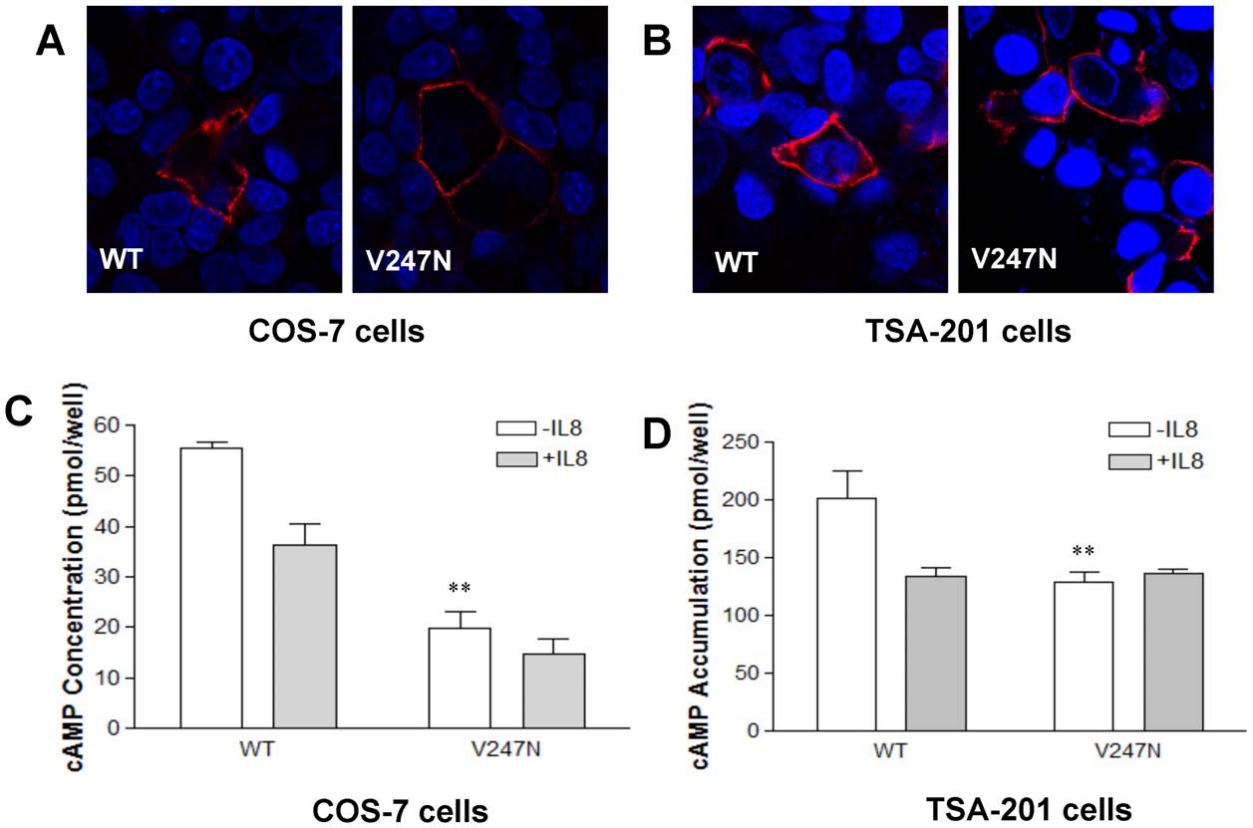
V247N mutant constitutively activates Gα_i_. (A and B) Surface expression of CXCR1 WT and V247N on COS-7 cells (A) and TSA-201 cells (B). Cells were incubated with mouse anti human CD128a (CXCR1) antibody at 4°C overnight. After washing three times, the cells were incubated with the secondary antibody (DyLight 549-conjugated goat anti-mouse IgG(H+L)) at RT for 1 hr and counterstained with Dapi. Expression of CXCR1 or the mutants was observed using a confocal microscope. The pink color represents the surface expression of CXCR1 WT or mutant with Dapi shown in blue. (C) Inhibition of forskolin-stimulated cAMP production in COS-7 cells. COS-7 cells were transfected with CXCR1 WT or V247N mutant, as well as Gα_i2_, Gβ_1_, and Gγ_2_ expression vector. IL-8 significantly inhibited forskolin-induced stimulation of cAMP formation in transfected cells whereas V247N mutation inhibited forskolin-induced cAMP levels even in the absence of IL-8. Transfected cells were used for determination of cAMP levels in the absence *(open bars)* or in the presence of IL-8 *(closed bars)*. The results shown are representative. (D) Inhibition of hCG-stimulated cAMP accumulation by V247N mutant. TSA-201 cells were transfected with CXCR1 WT or V247N mutant, as well as LH receptor expression vector. The TSA-201 cells were pretreated with 0.5 mM IBMX for 30 min followed by treatment with hCG (10^−7^ M) in the presence or absence of IL-8 (40 nM) for another 30 min. Following incubation with hCG in the presence or absence of IL-8, cells were lysed and intracellular cAMP measured using a RIA kit as described in “Experimental Procedures”. Data presented are the mean ± SEM for three assays with each condition performed in triplicate.

V247N is a robustly expressed mutant coupled to Gα_15_. Both CXCR1 WT and V247N mutant are expressed in transfected COS-7 cells (Fig. 7A) and TSA-201 cells (Fig. 7B). To test whether V247N is capable of constitutively activating Gα_i_ and inhibiting adenylyl cyclase, CXCR1 WT or V247N mutant were cotransfected with Gα_i2_, Gβ_1_, and Gγ_2_ in COS-7 cells. Receptor-mediated inhibition of forskolin-stimulated cAMP levels is a common approach to study the action of an inhibitory receptor on adenylyl cyclase. As shown in Fig. 7C, the addition of IL-8, in the presence of forskolin, led to a decrease in forskolin-induced cAMP levels in both CXCR1 WT and V247N mutant-transfected COS-7 cells by about 35% and 70%, respectively, compared with forskolin-induced cAMP levels in the absence of IL-8. In V247N-transfected COS-7 cells, the forskolin-stimulated cAMP production was significantly suppressed in the absence of IL-8 compared with CXCR1 WT transfected cells (*P*<0.01), suggesting that V247N constitutively activated Gα_i_-mediated signaling.

In order to further verify the ability of CXCR1 WT and V247N mutant to inhibit adenylyl cyclase, we utilized a protocol in which both the CXCR1 receptor and LH receptor were cotransfected. Cotransfection of the LH receptor, meant to stimulate adenylyl cyclase via Gα_s_ protein, allowed us to induce cAMP accumulation in those transfected cells. Again, it was found that in the transfected TSA-201 cells, LH receptor ligand hCG-stimulated cAMP production was inhibited in response to IL-8 in TSA cells expressing CXCR1 WT and V247N mutant (Fig. 7B, D). As shown in Fig. 7D, when CXCR1 WT or V247N is heterologously expressed in TSA-201 cells, IL-8 inhibited the hCG-stimulated accumulation of cAMP, indicating that both CXCR1 WT and V247N couple to Gα_i_. In addition, V247N mutant significantly suppressed hCG-stimulated cAMP accumulation (*P*<0.01) in the absence of IL-8, indicating that V247N mutant constitutively activated Gα_i_.

### Chemotaxis mediated by CXCR1 and its mutants

**Figure 8:**
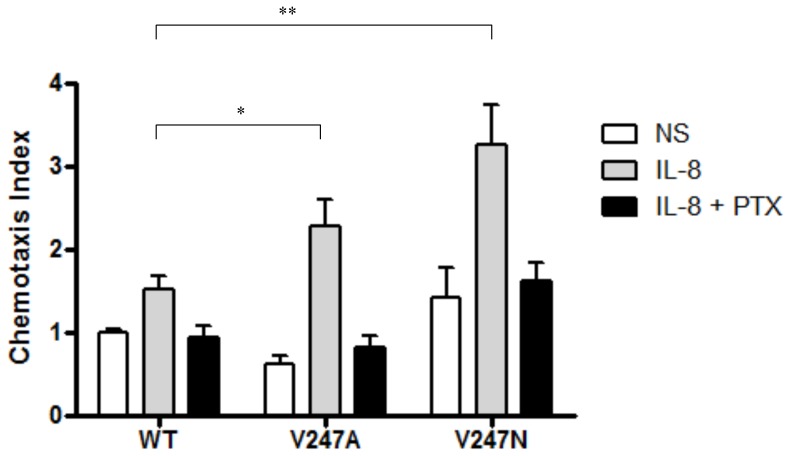
Chemotaxis assay of CXCR1 WT and its constitutively active mutants. Chemotaxis assays were performed as described in Materials and Methods, using HEK293 cells expressing constitutively active mutants (V247A and V247N) or the WT CXCR1. Data presented are the mean ± SEM for three assays with each condition performed in triplicate. *, *P*<0.05, V247A *vs.* WT (+IL-8). **, *P*<0.01, V247N *vs.* WT (+IL-8).

IL-8-induced chemotaxis via CXCR1 requires pertussis toxin-sensitive Gα_i_ protein [51]. To test whether CXCR1 and its constitutively active mutants can affect cell function, chemotaxis was measured in cells expressing the CXCR1 or its constitutively active mutants as well as Gα_i_, Gβ, Gγ, and PLCβ_2_. In the absence of IL-8 cells demonstrated disoriented, non-specific migration. We did not observe the increase in chemotaxis in non-IL-8-treated HEK cells expressing these mutants, suggesting that there were no differences in random migration between CXCR1 WT and constitutively active mutants. However, in the presence of IL-8, the cells expressing CXCR1 mutants (V247A and V247N) displayed enhanced migration than those expressing WT CXCR1, suggesting that the constitutively active mutants respond to IL-8 above and beyond their constitutive activity. Both CXCR1 and its constitutively active mutants induced chemotaxis in response to IL-8 (Fig. 8). Compared to the WT receptor, the constitutively active mutants demonstrated a 40% and 100% increase in maximal migration index for V247A and V247N, respectively, in response to IL-8. PTX blocked chemotaxis mediated by the WT receptor and constitutively active mutants, suggesting that Gα_i_ signaling was required (Fig. 8).

## Discussion

In this study we attempted to identify the role of two amino acid residues on CXCR1, L128 in TM3 and V247 in TM6, in Gα_i_ and Gα_15_ coupling. Although our results suggest that both L128 and V247 are critical amino acid residues involved in Gα_15_ protein coupling in transfected COS-7 cells, these two amino acid residues play distinct roles in G protein coupling. L128, for example, is involved in the coupling of the receptor to Gα_15_ and to Gα_i_. Although mutations in L128 did not affect either its expression or its binding to IL-8, substitution of this residue caused either the loss of or dramatically reduced response to IL-8 that resulted from impaired interaction between the receptor and G proteins. V247, on the other hand, is associated with maintaining the inactive conformational structure of CXCR1 so that selective mutation of residue V247 either transformed the receptor from inactive to active conformation or stabilized the receptor in its active state, resulting in the constitutive activation of the receptor.

Detailed functional studies of constitutively active CXCR1 mutations, combined with homology modeling from the active and inactive rhodopsin as well as H2A receptor crystal structures serving as templates, led to important insights into the mechanism of activation, even in the absence of crystal structure of GPCRs in active state. Our finding that substitutions of V247 in transmembrane helix 6 of CXCR1 caused constitutive activation of the Gα_15_ and Gα_i_ signaling pathway may provide new insights into the specific process by which chemokine binding triggers G protein activation. The role for IL-8 in CXCR1/CXCR2-mediated neutrophil recruitment in chronic airway inflammatory diseases, including COPD and cystic fibrosis, has been delineated. CXCR1 blockade retards viability of breast cancer stem cells and reduces metastasis [52]. Inhibition of human melanoma growth by CXCR1 antagonist has been reported [53]. Constitutive activation of CXCR1 may provide a rapid and sensitive readout for GPCR signaling and may facilitate screening and/or developing novel IL-8 antagonists for the treatment of neutrophil-mediated diseases.

Leu3.43 is highly conserved in type A GPCRs and is critical in activation of several GPCRs [43]. Even when bound to IL-8, L128A and L128R (obtained by introducing the amino acid Ala or Arg at TM3.43) either totally impaired their coupling to Gα_15_ or dramatically impaired their coupling to Gα_i_ despite their ability to bind to their ligand with high affinity. This implies that L128 is critical for Gα_15_ and Gα_i_ coupling and that there are discrete steps in binding and signaling of IL-8 with its receptor. Recently, a two-site multistep model of how IL-8 binds and activates CXCR1 was proposed. In this model, CXC motif functions as a conformational switch that couples Site-I and Site-II interactions for CXCR1. This coupling is critical for high affinity binding but regulates activation differently [22]. Our results with mutations at residue Leu128 support these hypotheses and suggest that this single amino acid residue may not be involved in ligand binding but may be involved in the subsequent receptor activation.

V247 residue corresponds to Baldwin location on TM6.40. The identification of CXCR1 mutants with constitutive activity points to the critical role of this amino acid residue in G protein coupling and receptor activation. It was proposed that the function of the inactive, constrained GPCR conformation is to conceal key cytoplasmic peptide sequences and thus prevent them from interacting with G proteins [31], [54]. Disruption of the constraints and stabilization of the receptor to an active conformation would expose the cytoplasmic residues that can bind and activate the relevant G proteins [31], [54]. Thus, amino acid V247 is probably critical in maintaining CXCR1 in an inactive state. In line with our findings, it is likely that the constraint of intramolecular bond stabilizing CXCR1 in an inactive state is disrupted by substitution of V247 with A or N which would switch the transmembrane domains to convert the receptor to an active state.

Recent observations in the structure of light-activated rhodopsin has demonstrated that the movement of cytoplasmic ends of TM5 and TM6 away from the receptor core opens up a cleft in the center of the helix bundle, thereby allowing the carboxy terminus of a G protein to bind [55]. Comparison of the agonist- and inverse agonist-bound β2AR structure also revealed the largest change to be in TM6, with the outward movement of the cytoplasmic end of TM6 and rearrangements of TM5 and TM7 that are remarkably similar to those observed in active form of rhodopsin [30], [34], [55], [56], [57], [58], [59]. Beta2AR structure showed weak interactions between the cytoplasmic ends of TM3 and TM6 which may account for the relatively high basal activity and structural instability of the β2AR [60]. TM6 is thought to be one of the major players in the signaling mechanism and disruption of its structure would likely affect G protein binding and activation [29], [55], [61]. Our study on agonist-independent constitutive activity of CXCR1 mutants (V247A and V247N) on TM6 and its signaling provides insights into the process of agonist binding and activation of GPCRs.

The amino acid residue located at TM6.40 might be critical in the stabilization of the receptors coupled to various G proteins such as Gα_s_, Gα_15_, Gα_i_ or G_t_ in the inactive state. Mutations of TM6.40 in opsin (Met257), TSH receptor (Leu629), muscarinic receptor (Ile447), and histamine H_1_ receptor (Ile420) also result in constitutive activation [39], [40], [43], [44]. Although the particular residue might be different at this site (Met257 in bovine rhodopsin, Leu629 in TSHR, Ile447 in the muscarinic receptor, and Val247 in CXCR1), the residue at TM6.40 appears to participate in G protein coupling and receptor activation as a result of closely related deviations from regular α-helical structure and similar tertiary structures. In fact, replacement of TM6.40 in several different GPCRs (Gt-coupled rhodopsin, Gα_s_-coupled TSHR, and Gα_i_/Gα_15_-coupled CXCR1) results in constitutive activity of the receptors coupled to specific G proteins (Gα_s_, G_t_, Gα_i_ or Gα_15_). This suggests that the amino acid residue located at TM6.40 is critical for the stabilization of the receptors in the inactive state, which is an intrinsic feature of the receptors and independent of the G protein type. Structural modification of this single amino acid residue affects GPCR-G protein interaction and receptor activation.

Both V247A and V247N demonstrated high efficiency in coupling to Gα_15_. Without ligand, mutants V247A and V247N were stabilized in the active state. However, ligand binding caused certain responses including elevated IP accumulation, which might result from conformational changes caused by receptor binding to its ligand, IL-8. There is strong evidence that GPCRs may exist in multiple ligand-specific conformational states [31], [54]. In the multistate model, the receptor is proposed to alternate between multiple active and inactive conformations. A recent publication on the agonist-bound adenosine A_2A_ receptor structures revealed an intermediate conformation between the inactive and active states to support this model [28]. In agreement with this, the observation that both V247A and V247N CXCR1 mutants with distinct K_d_ in this study are constitutively active strongly supports the concept that more than one active receptor state can exist.

Transmembrane signaling through CXCR1 plays a role in many neutrophil functions such as chemotaxis [4]. Many of these signaling pathways have emerged from the studies using cell lines transfected with receptors or related cellular components. With regard to our use of COS-7 cells, most of the reconstituted components, including Gα_i2_, Gβ_1_, Gα_15_ and PLCβ_2_ in these cells, are likely to be found in neutrophils or related myeloid cells where CXCR1 is expressed [19]. Therefore, both the Gα_15_- PLCβ_2_ and the Gα_i_-Gβγ-PLCβ_2_ pathways are likely to be active *in vivo*. In addition, chemotactic responses in many leukocytes require activation of Gα_i_, as indicated by their sensitivity to inhibition by PTX. In agreement with this, our data indicated that chemotaxis mediated by CXCR1 or its mutants is completely abolished in transfected cells pretreated with PTX. Therefore, the increase in chemotaxis in response to IL-8 observed in cells expressing constitutively active mutants V247A and V247N can be attributed to the active signaling through these mutants.

In summary, the two residues in TM3 and TM6 on CXCR1 that correspond to L128 (TM3.43) and V247 (TM6.40), respectively, play distinct roles in receptor activation. Changes in conformational structure by substituting L128 impaired the interaction between the receptor and G proteins without affecting ligand binding. The substitution of V247 impaired the receptor constraint and stabilized an active conformation. The active CXCR1 mutants demonstrated increased chemotaxis in response to IL-8 in a PTX-sensitive manner. These findings regarding the constitutively active form of CXCR1 may contribute to our knowledge of the activation of CXCR1 and may be potentially useful for designing and screening CXCR1-specific antagonists which may serve as targets for prevention and therapy of certain neutrophil-mediated inflammatory diseases such as COPD.
